# Tunnel construction worker safety state prediction and management system based on AHP and anomaly detection algorithm model

**DOI:** 10.1016/j.heliyon.2024.e36450

**Published:** 2024-08-21

**Authors:** Yuhao Cao, Bai Yun

**Affiliations:** a School of Computer Science and Technology, Wuhan University of Science and Technology, Wuhan 430065, Hubei, China; b College of International Studies, National University of Defense Technology, Nanjing 210039, China; c Basic Education College, National University of Defense Technology, Changsha 410073, China

## Abstract

Tunnels represent complex, high-risk, and technically demanding underground construction projects. The safety of construction workers in tunnels is influenced by various factors, including physiological indicators, tunnel dimensions, and internal environmental conditions. Analyzing safety based solely on static factors is inadequate for modern tunnel engineering safety management requirements. To address this challenge, this paper provides a comprehensive analysis of factors impacting safety and employs the Analytic Hierarchy Process (AHP) to identify seven significant factors with high importance: body temperature, heart rate, internal temperature, internal humidity, CO concentration, chlorine concentration, and the relative positioning of personnel. Considering these factors essential for assessing worker safety, we introduce a novel model named Tunnel-APH-AD. For training models aimed at anomaly detection, we performed data augmentation and utilized four distinct machine learning models. Additionally, ensemble learning techniques were applied to aggregate the predictions from individual models, thereby enhancing the effectiveness of detecting safety states for tunnel workers. We also evaluated the performance of these models on out-of-distribution (OOD) samples to test their robustness and generalizability. The experimental results indicate that, under similar ventilation and tunnel conditions, the ensemble learning model exhibits superior overall performance compared to individual models, underscoring the effectiveness of model combination in improving the accuracy and reliability of safety alerts. Through experimental validation, this study provides interpretable, scalable, and scientifically generalized applications of machine learning theories in systems for tunnel construction worker safety alerts. These findings contribute to advancing safety management practices in tunnel engineering, enabling proactive and effective measures to mitigate potential risks and ensure the well-being of workers.

## 0 Introduction

Tunnel construction is a high-risk and hazardous industry, where safety accidents frequently occur, posing significant threats to the well-being and physical integrity of construction workers [1], [2], [3]. These accidents can lead to irreparable casualties and substantial property loss. Therefore, it is of utmost importance to develop a scientific and effective approach [4] to predict and manage the safety state of tunnel construction workers.

While the severe consequences of tunnel construction accidents are acknowledged, the critical need to develop scientific and effective methods for predicting and managing the safety status of construction personnel is emphasized. This focus is warranted as the safety of personnel is not only a matter of preventing accidents but also ensuring continuous operational safety and minimizing potential risks. Predictive safety management is particularly vital as it allows for proactive measures that can preemptively address conditions before they escalate into accidents. While other preventive measures such as structural assessments, material quality control, and compliance with safety regulations are undoubtedly effective, they often address broader safety aspects without the specific, real-time focus required to directly safeguard the workers on a day-to-day basis. Predicting and managing the conditions and states of construction personnel takes into account the dynamic and unpredictable nature of the human elements in construction sites, which are often the first indicators of potential risks.

With the rapid development of information technology, researchers have increasingly explored the use of computer technology to address safety concerns in tunnel construction. This paper proposes a Tunnel Construction Worker Safety State Prediction and Management System based on the Analytic Hierarchy Process (AHP) [5], [6], [7] and Anomaly Detection Algorithm Model [8], [9], [10]. This system collects various data sources, including personal information of construction workers and environmental data, and utilizes the AHP model to analyze the importance and weights of the data, facilitating the identification of potential safety hazards. Additionally, the system incorporates an anomaly detection algorithm model to promptly detect and predict potential safety risks [11], providing more accurate management recommendations and decision support.

Traditionally, safety management in tunnel construction [12], [13] heavily relies on static factors and manual inspections. These approaches are limited in their ability to capture the dynamic nature of safety risks [14]. However, with advancements in technology, researchers have started exploring data-driven approaches to enhance safety management. Some studies have focused on utilizing machine learning techniques for safety prediction [15], while others have employed sensor technologies and wearable devices to collect real-time data on workers' physiological indicators [16]. Despite these efforts, there is still a need for an integrated approach that effectively combines different factors to provide a comprehensive safety assessment.

The existing methods in tunnel construction safety management face several challenges. First, the reliance on static factors limits the ability to account for real-time changes and dynamic hazards [17]. Second, the integration of multiple data sources, including workers' physiological indicators and environmental conditions, is often lacking [18]. Third, accurately identifying and predicting potential safety risks requires sophisticated analytical techniques. Finally, efficient decision support systems [19] that provide timely recommendations to managers are necessary.

To address these challenges, this paper proposes a comprehensive approach that combines the Analytic Hierarchy Process (AHP) and anomaly detection algorithms. The AHP model allows for the analysis of various data sources, assigning importance weights to different factors, and facilitating the identification of potential safety hazards [20]. The anomaly detection algorithm model utilizes machine learning techniques to detect abnormal patterns and predict safety risks based on the collected data. By integrating these approaches [21], the proposed system provides a more accurate and timely assessment of the safety state of tunnel construction workers. The contributions of this paper can be summarized as follows:

• The combination of AHP and anomaly detection algorithms enables an effective prediction and management of tunnel construction worker safety state. This integrated approach allows for a comprehensive assessment of safety risks and provides a foundation for proactive safety measures.
• We propose a data-driven approach, leveraging various data sources including workers' physiological indicators and environmental conditions. By considering multiple factors, the proposed method offers a more comprehensive, scientific, and accurate assessment of the safety state of construction workers.
• We design a web-based management system, empowering managers to monitor the safety state of construction workers in real-time. The system provides a user-friendly interface that enables timely decision-making and the implementation of necessary safety measures.

## 1 Analysis of tunnel construction site data and influencing factors

In this section, we focus on the Jinkouhe Tunnel Project in Leshan [22], Sichuan as the background. There are numerous factors influencing the safety of tunnel construction personnel. From a time-series perspective, these factors include body temperature [23], [24], heart rate, gas concentrations inside the tunnel, temperature, and humidity. From a spatial perspective, factors include the distance to the nearest safe exit for construction personnel and the location of sensors [25]. The choice of these specific indicators over others is crucial for several reasons. Firstly, body temperature and heart rate are primary indicators of a worker's health status. Changes in body temperature can indicate heat exhaustion or hypothermia, both of which are critical conditions that can occur in tunnel environments. Similarly, monitoring heart rate can provide immediate data regarding a worker's exertion level or stress, which is particularly important in avoiding cardiovascular incidents. Secondly, gas concentrations, temperature, and humidity within the tunnel are selected due to their direct impact on both the immediate safety and long-term health of the personnel. High levels of toxic gases can lead to asphyxiation or poisoning, while extreme temperatures and inappropriate humidity levels can contribute to discomfort and health risks, impacting workers' concentration and efficiency. Furthermore, the spatial factors such as the distance to the nearest safe exit and the location of sensors are crucial for emergency response planning. These factors ensure that personnel can quickly evacuate in case of an emergency and help in efficient monitoring and rapid response to any incidents within the tunnel. Therefore, in this chapter, we employ the Analytic Hierarchy Process (AHP) to study and analyze the safety influencing factors at the tunnel construction site, with significant factors shown in the diagram. In Sections 3.1 and 3.2, this paper analyzes the influencing factors from both the temporal and spatial dimensions. This comprehensive approach allows for a detailed understanding of how these factors interplay and influence overall safety, enabling better prevention strategies and risk management at the construction site.

### 1.1 Temporal characteristics of data

Regarding body temperature [26], heart rate, tunnel temperature, tunnel humidity [27], CO concentration [28], and chlorine concentration [29], they all exhibit patterns that vary over time. This paper identifies them as time-series data.

#### 1.1.1 Body temperature and heart rate

During actual operations, symptoms such as fatigue, mental confusion, pose a threat to the safety of construction workers. Body temperature and heart rate directly reflect the physiological state of individuals. Temperature screenings should be conducted for all personnel entering the construction site to ensure epidemic prevention and control [30]. Individuals with a temperature ≥37.3 °C should be immediately isolated and observed in designated dormitories. Heart rate parameters are highly sensitive to temperature changes, making them easy to measure and calculate, with minimal time consumption [31]. The normal heart rate for adults is 60-100 beats per minute. During enclosed space operations, if a worker's heart rate exceeds the normal range, work should be stopped immediately to prevent impairments to the worker's physical and mental functions [32], thus avoiding severe work accidents.

#### 1.1.2 Tunnel temperature and humidity

Numerous factors influence the temperature and humidity inside the tunnel [33], including mechanical ventilation, natural air, groundwater, diurnal and seasonal temperature and humidity variations outside the tunnel, heat released by construction machinery, geothermal energy, air pressure, wind direction, and wind speed changes. Temperature and humidity not only reflect the working conditions of construction personnel but also indicate the safety of the tunnel structure.

Excessive humidity inside the tunnel results in high water vapor content in the air, making it difficult for sweat to evaporate from the body, hampering heat dissipation. The high humidity environment increases friction between our body's sweat and clothing. High temperature and high humidity have adverse effects on personnel working inside the tunnel, reducing work efficiency [34] and potentially endangering lives, while also increasing the occurrence of safety accidents. When the tunnel temperature decreases within a certain range, it directly affects the physiological and mental state of individuals. Low temperatures can lower the body's immune system, leading to various illnesses such as colds and asthma.

#### 1.1.3 Tunnel gas

Carbon monoxide (CO) combines with hemoglobin in the blood [35], forming stable carboxyhemoglobin, which leads to suffocation in humans. When the concentration exceeds 100 PPM in the environment, individuals gradually experience symptoms such as dizziness and fatigue. Chlorine gas is a toxic gas with a yellow-green color and is primarily inhaled through the respiratory tract. Chlorine gas dissolves in liquids, forming hypochlorous acid and hydrochloric acid. Hypochlorous acid can cause inflammation [36] of the skin and mucous membranes, leading to coughing symptoms. If the condition worsens, it can cause pulmonary edema, disrupt the circulatory system, and ultimately result in fatality.

### 1.2 Spatial characteristics of data

**Figure 1:**
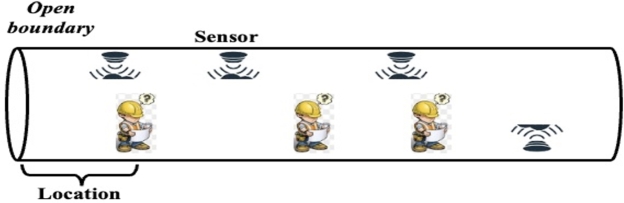
Tunnel abstract diagram.

The tunnel abstract diagram shown in Fig. 1 represents the spatial characteristics considered in this paper. And the specific characteristics are demonstrated below. Firstly, the relative positions of construction workers to the tunnel entrance are of vital importance to their safety during tunnel construction. Different construction positions may pose varying levels of danger and present different levels of difficulty for search and rescue operations [37]. It is necessary to track the location of each individual within the tunnel as they move or work. The installation of personnel positioning devices requires determining the coordinates of each base station, with the tunnel entrance as the starting point. By knowing the positions of personnel within the tunnel, the distance between construction workers and the tunnel entrance can be calculated. Tracking the distance of individuals inside the tunnel from the entrance allows for real-time statistics on the total number of people in the tunnel and the distribution of personnel in various areas. Accurate determination of the distance between individuals and the tunnel entrance facilitates rescue operations in case of safety emergencies. This paper provides a formal definition for the concept of relative position and proposes regional localization of construction workers, with the distance from the tunnel entrance considered as an absolute position. The reference position is determined based on on-site construction conditions, and in this paper, the reference position is defined as 100 meters [38]. For example, if a construction worker is 200 meters away from the tunnel entrance, their relative spatial position would be 2. Decimal numbers are rounded using the rounding method.

Secondly, the positions of sensors are also considered. Sensors can collect gas data inside the tunnel. When there is only one sensor, the continuous data collected can be understood as time-series data. However, it is evident that deploying only one sensor inside the tunnel is not feasible. Therefore, when multiple sensors are present, spatial characteristics come into play [39], as shown in the figure. This is also the reason for considering the use of graph convolutional neural networks, as they need to learn dependency information in the spatial domain.

### 1.3 Influence factor selection based on AHP algorithm

The AHP method was applied to study and analyze the structural diagram of safety influence factors for the Jin Kou He Tunnel Construction Project in Leshan, Sichuan [40]. When using safety early warning indicator data in this model, quantifiable factors should be taken into consideration [41]. Firstly, it is necessary to identify the weights of the influential factors from personnel factors, environmental factors, and management factors, which are illustrated in Table 1.

**Table 1:** Intermediate Judgment Matrix.

Criterion	Personnel Factors	Environmental Factors	Management Factors
Personnel Factors	1	0.25	2
Environmental Factors	4	1	8
Management Factors	0.5	0.125	1

Table 2 presents the weight calculation results using the Analytic Hierarchy Process (AHP). The weight calculation results from the AHP method indicate that the personnel factors have a weight score of 0.1921, the environmental factors have a weight score of 0.7133, and the management factors have a weight score of 0.0909. Therefore, this study initially considers the influence of personnel factors and environmental factors.

**Table 2:** Intermediate AHP Analysis Results.

Criterion	Eigenvector	Weight	Max Eigenvalue	CI Value
Personnel Factors	0.7937	0.1921	-	-
Environmental Factors	3.1748	0.7133	3	0
Management Factors	0.3969	0.0909	-	-

The calculation results of the Analytic Hierarchy Process (AHP) indicate that the maximum eigenvalue is 3.0. Referring to the RI table, the corresponding RI value is found to be 0.525. Therefore, the consistency ratio (CR) is calculated as CI/RI=-0.0<0.1, passing the consistency test.

## 2 Method

### 2.1 Problem definition

In this paper, data from tunnels is collected, primarily sourced from N sensors, constituting multivariate spatio-temporal sequence data [42]. The data collected by the sensors is defined as $S_{train}=[S_{train_{1}},...,S_{train_{n}}]$, which is used to train our method. At each time point t, the sensor values $s(t)\in R^{N}$ form an N-dimensional vector, representing the values of our N sensors. Following the common unsupervised anomaly detection approach, the training data is assumed to only include normal data. Our goal is to detect anomalies in the test data, which come from the same N sensors but at a separate set of Ttest time scales: the test data is represented as $S_{test}=[S_{test_{1}},...,S_{test_{n}}]$. The output of our algorithm is a set of binary labels for Ttest, indicating whether each test time point is an anomaly, i.e., a(t)∈{0,1}, where a(t) = 1 denotes an anomaly at time t, and a(t) = 0 denotes normalcy.

### 2.2 Overview

**Figure 2:**
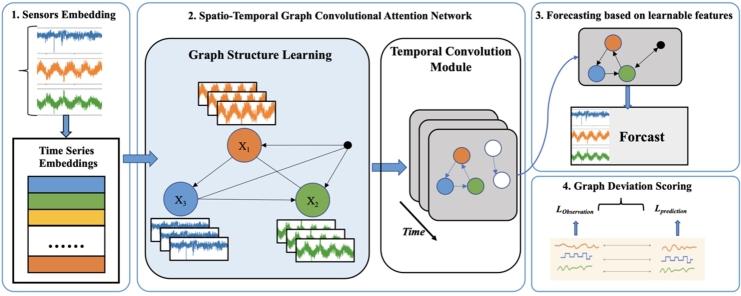
The overview of our model Tunnel-APH-AD, which is composed of three key components, containing Sensors Embedding, Spatio-temporal Graph Convolutional Attention Network, Forecasting Based on Learnable Features, and Graph Deviation Scoring.

The proposed model aims to consider using a graph-based approach [43] to learn the temporal sequence data acquired by sensors at different locations. The spatio-temporal graph representation effectively captures the temporal and spatial relationships between the data. It then identifies and explains the deviations existing between the learned patterns. The model mainly consists of four components, which will be introduced one by one. The details of our model are illustrated in Fig. 2.

In many sensor data settings, different sensors can exhibit highly diverse characteristics that can be intricately interconnected [44]. For instance, in a tunnel setting, sensors are placed at the ceiling with a spacing of five meters. Let's assume there are 10 sensors, and the data measured by each sensor follows a temporal sequence format. However, there exists implicit information regarding the spatial relationships among the sensors, implying a certain spatio-temporal aspect. Therefore, ideally, the goal of this paper is to devise a novel and flexible approach to describe the correlations between the data collected by each sensor, capturing diverse patterns in a multidimensional manner. For each sensor's data, we introduce a set of vectors to represent its characteristics, as shown in the following equation:

(1) $$v_{i}\in R^{d},\;\mathrm{for}\;i\in \{1,2,\cdots ,N\}.$$

These embedding vectors are randomly initialized and trained along with the entire model. The similarity between the embeddings, denoted as $V_{i}$, indicates the relationships between the underlying patterns in the data. In the proposed model, these embeddings serve two functions:

(1) They are used for spatio-temporal information learning, enabling the identification of information with temporal and spatial correlations.
(2) In the attention mechanism module of the model, they facilitate attention over neighbors in a consistent manner, mitigating the heterogeneous effects of different types of sensors.

Additionally, in this subsection, after collecting the data, a normalization operation is applied to eliminate the influence of dimensionless units. The normalization formula is as follows:

(2) $$X_{norm}=\frac{X-X_{min}}{X_{max}-X_{min}}.$$

### 2.3 Spatio-temporal graph convolutional attention network

The overall framework of this paper is illustrated in the figure below. As described earlier, the objective of this study is to capture the spatio-temporal dependencies among data collected by different sensors. To achieve this, a spatio-temporal graph convolutional neural network (GCN) is employed. We utilize a directed graph, where the nodes represent sensors, and the edges represent the spatio-temporal dependencies between sensors. An edge from one sensor to another captures the spatio-temporal behavior between the two sensors. The choice of a directed graph is made because the spatio-temporal dependencies between sensors are not symmetric. To represent this directed graph, we employ an adjacency matrix *A*, where $A_{ij}$ represents the directed edge from the node of sensor *i* to the node of sensor *j*.

Considering the dependencies among sensor information, we construct an array list. This prior information enables us to flexibly and naturally select a set of candidate relations $C_{i}$ for each sensor *i*. In other words, it determines which sensors may form spatio-temporal dependencies with a particular sensor.

(3) $$C_{i}\subseteq \{1,2,\cdots ,N\}\setminus \{i\}.$$

In the absence of prior information, the candidate relations for sensor *i* include all sensors except itself. To determine the spatio-temporal dependencies between sensor *i* and other sensors, we calculate the similarity between the embedding vector of node *i* and the embeddings of its candidates $j\in C_{i}$.

(4) $$e_{ji}=\frac{{v_{i}}^{⊤}v_{j}}{\left\|v_{i}\|\cdot \|v_{j}\right\|}\;\mathrm{for}\;j\in C_{i},A_{ji}=1\{j\in \mathrm{TopK}(\{e_{ki}:k\in C_{i}\})\}.$$

In essence, the procedure initiates by calculating $e_{ji}$, which represents the normalized dot product of the embedding vector corresponding to sensor *i* and the candidate relation *j* within the set $C_{i}$. Subsequently, the methodology involves the selection of the top-k such normalized dot products. It is pertinent to note that TopK signifies the indices of the top-k values within its input array, specifically the normalized dot products. The parameter *k* is adjustable by the user, allowing for the customization of the sparsity level of the resulting matrix. Following this, we delineate the structure of our spatio-temporal graph attention-based model, which incorporates the aforementioned adjacency matrix *A* in its framework.

### 2.4 Forecasting based on learnable features

Interpretability in machine learning is one of the important research directions in the field. From this perspective, we consider how to study the generation of anomalous phenomena and provide effective interpretability for abnormal occurrences in the field of tunnel safety engineering. Therefore, we hope our model can answer the following questions:

(1) Which sensors' data deviates from normal behavior and exhibits anomalous values? (2) In which specific aspects does the behavior of a particular sensor deviate from normal?

After employing the spatio-temporal graph network for feature encoding, we obtain high-dimensional features of the raw data. We adopt a prediction method based on learned features, which is conceptually simple: using past historical information to predict future information. This can be approximated as a Markov decision process. In the context of this paper, numerous sensors are installed inside the tunnel. Using the proposed model, we predict the behavior states of each sensor in the short term. This enables tunnel construction personnel to quickly and easily identify abnormal states. Additionally, tunnel construction personnel can swiftly understand the observed behaviors of different sensors and gain insights into what specific conditions may compromise tunnel safety.

Despite the apparent focus on detecting anomalies, the ultimate goal is not solely to identify outlier sensor data but to understand the underlying risks associated with these anomalies. When a sensor's data deviates from expected patterns, it signals a potential hazard; for example, a spike in gas concentration could indicate a leak, while erratic readings from structural sensors may suggest stability issues within the tunnel. Moreover, while anomalies in temperature and heart rate sensors might directly indicate adverse physical conditions affecting the construction workers, it is imperative to further investigate these physiological parameters. Abnormal readings could stem from various sources beyond immediate health risks to workers, such as equipment malfunction or environmental influences that do not directly harm human health but could lead to unsafe working conditions. For instance, an elevated heart rate could be influenced by external stressors unrelated to physical exertion, such as psychological stress or ambient temperature extremes.

Input Layer: In summary, at time t, we employ the concept of a sliding window with a window size of w to predict the future based on historical spatio-temporal sequence data. Therefore, our model takes the following as input:

(5) $$x^{\left(t\right)}:=\left[s^{\left(t-w\right)},s^{\left(t-w+1\right)},\cdots ,s^{\left(t-1\right)}\right]\in R^{N\times w}.$$

Target Output: The output that our model needs to predict is the sensor data at the current time point, i.e., s(t).

**Feature Extraction Layer:** To learn spatial information on different sensor locations and their relationships, we propose a graph-based learner in this paper. Based on the learned graph structure information, we aggregate the node information represented by each sensor together. Considering the importance of learning better relational features, we incorporate the embedding vector *v* of sensor data during the data input stage, similar to the role of a position vector. This vector can characterize different behaviors resulting from data collected by sensors at different locations. Therefore, we compute the aggregated representation $z_{i}$ of sensor *i* as follows:

(6) $$z_{i}^{\left(t\right)}=\text{ReLU}\left(\alpha_{i,i}Wx_{i}^{\left(t\right)}+\sum_{j\in N(i)}\alpha_{i,j}Wx_{j}^{\left(t\right)}\right),$$

where $x(t)\in R^{w}$ is the input feature of node i, $N(i)=j,|,A_{ji}>0$ is the set of neighbors of node i obtained from the learned adjacency matrix A, and $W\in R^{d\times w}$ is the trainable weight matrix that applies a shared linear transformation to each node. The attention coefficients $\alpha_{ij}$ are combined as follows:

(7) $$g_{i}^{\left(t\right)}=v_{i}⊕Wx_{i}^{\left(t\right)}\;\pi \left(i,j\right)=\text{LeakyReLU}\left(a^{⊤}\left(g_{i}^{\left(t\right)}⊕g_{j}^{\left(t\right)}\right)\right)\alpha_{i,j}=\frac{\exp\left(\pi \left(i,j\right)\right)}{\sum_{k\in N(i)\cup \left\{i\right\}}\exp\left(\pi \left(i,k\right)\right)},$$

In the given context, ⊕ symbolizes the operation of concatenation. Thus, $g{\left(t\right)}_{i}$ effectuates the concatenation of the sensor embedding $v_{i}$ with the transformed feature $Wx{\left(t\right)}_{i}$, where *a* denotes the learned coefficient vector pertinent to the attention mechanism. The computation of the attention coefficients employs LeakyReLU as the non-linear activation function. Subsequently, the normalization of these attention coefficients is achieved through the application of the softmax function, as delineated in Equation (8).

Similarly, in the temporal dimension, we perform convolution operations to sequentially aggregate more local feature information. The formula is as follows:

(8) $$T^{l+1}=\text{ReLU}\left(\Phi ⁎\left(\text{ReLU}\left(T^{l}\right)\right)\right),$$

where + denotes the representation at the next time step, ⁎ represents the standard convolution operation with kernel size *k*, ReLU is the activation function. The temporal convolutional network updates the node features by incorporating information from adjacent time steps, enabling it to effectively capture temporal dynamics.

**Output Layer:** From the aforementioned feature extraction layer, we obtain the feature representations of all sensors represented as nodes in the graph, denoted as $z{\left(t\right)}_{1},\ldots ,z{\left(t\right)}_{N}$. Next, we perform element-wise multiplication with the corresponding spatio-temporal sequence feature vectors and apply a fully connected layer to obtain a feature vector of dimension *N*. This allows us to predict the sensor value vector at the next time step, s(t).

(9) $${\overset{ˆ}{s}}^{\left(t\right)}=f_{\theta }\left(\left[v_{1}\circ z_{1}^{\left(t\right)},\cdots ,v_{N}\circ z_{N}^{\left(t\right)}\right]\right).$$

For the loss function, we utilize the Mean Squared Error (MSE) to measure the discrepancy between the predicted values and the ground truth. The loss function can be formulated as follows:

(10) $$L_{\mathrm{MSE}}=\frac{1}{T_{\mathrm{train}}-w}\sum_{t=w+1}^{T_{\mathrm{train}}}{\left|{\overset{ˆ}{s}}^{\left(t\right)}-s^{\left(t\right)}\right|}_{2}^{2}$$

### 2.5 Inference module

After training the model, unlike traditional offline inference, the inference module in this paper adopts the idea of transfer learning. It allows continuous model updates based on new samples, thereby improving the model's generalization ability and better handling out-of-distribution (OOD) samples. This enables the model to adapt better to diverse environments.

## 3 Experiments

### 3.1 Dataset

The experiments in this study were conducted entirely in the real-world setting of the Jinkouhe Tunnel Project in Leshan, Sichuan. However, due to the scarcity of labeled datasets in the real world, we employed crowdsourcing to simulate the real-world tunnel scenarios. We integrated digital and physical elements to control and monitor the real-time state of the tunnel system. Finally, we combined the collected real-world dataset with the simulated dataset. For convenience, we named the dataset SCTDataset [45]. Table 3 provides an example showcasing the data.

**Table 3:** Sensor Data and Statistical Features.

Sensor Type	Maximum	Minimum	Standard Deviation	Reference Value
Body Temperature	37.1	35	0.65	36-37.2 °C
Heart Rate	144	50	14.25	60-100 beats/min
Environmental Temperature	20.6	7.3	2.6042	5-20 °C
Environmental Humidity	76	48	11.4427	45-65%
CO Monitoring	26.8	4.1	5.3397	≤24 PPM
CL2 Monitoring	2.23	0.68	0.3715	≤2 PPM
Relative Position	11	1	2.5089	-

### 3.2 Experimental setup and evaluation metrics

We evaluate the performance of our method and baseline models using precision (Precision), recall (Recall), and F1 score (F1) [46] on the test dataset. Their formulas are demonstrated as follows:

(11) $$\text{Precision}=\frac{TP}{TP+FP},$$

where Precision is defined as the ratio of True Positives (*TP*) to the sum of True Positives and False Positives (*FP*), quantifying the model's accuracy in predicting positive instances.

(12) $$\text{Recall}=\frac{TP}{TP+FN},$$

where Recall, also known as Sensitivity, measures the proportion of actual positives that are correctly identified by the model, calculated as the ratio of True Positives to the sum of True Positives and False Negatives (*FN*).

(13) $$F1=2\cdot \frac{\text{Precision}\times \text{Recall}}{\text{Precision}+\text{Recall}},$$

where the F1 Score is the harmonic mean of Precision and Recall, offering a balance between them and providing a comprehensive measure of the model's accuracy, especially in cases where the class distribution is imbalanced. This metric harmonizes the trade-offs between Precision and Recall, yielding a singular measure of performance that accounts for both the avoidance of false positives and the necessity of capturing all relevant instances.

In this paper, TP (True Positives), TN (True Negatives), FP (False Positives), and FN (False Negatives) denote the numerical counts of true positives, true negatives, false positives, and false negatives, respectively. It warrants emphasis that the dataset under consideration exhibits an imbalance, a condition that substantiates the choice of these metrics as being apt for scenarios characterized by imbalanced data [47]. For the purpose of identifying anomalous instances, a threshold is established predicated on the apex anomaly score observed within the evaluation dataset. Consequently, during the phase of testing, any temporal interval registering an anomaly score that surpasses this threshold is classified as anomalous.

### 3.3 Comparative experiment

We compare our model with PCA, KNN, OCSVM, LSTM, and Auto-Decoder models, and their respective performance metrics on the SCTDataset are shown in Table 4.

**Table 4:** Performance Evaluation of Models.

Models	Precision	Recall	F1-Scores
PCA	29.21	20.63	0.26
KNN	7.89	7.89	0.08
OCSVM	77.89	76.45	0.64
LSTM	93.23	61.21	0.73
Auto-Decoder	96.54	64.32	0.78
Ours	99.32	71.21	0.83

As shown in Table 4, we demonstrate the advantages of our model compared to the baseline models in terms of Precision, Recall, and F1-Score on the SCTDataset. Our proposed model achieves a precision of 99.32%, which is nearly 3% higher than the best-performing baseline. Furthermore, our model outperforms the second-best baseline model by almost 7% in terms of Recall. In terms of F1-Score, our model consistently outperforms the baseline models, surpassing the second-best baseline model by 0.05. In conclusion, our model exhibits excellent performance on the dataset, which is of paramount importance in real tunnel scenarios.

### 3.4 Ablation study

The ablation experiments are highly necessary to investigate the importance of each component module in our model. In order to assess the significance of each component, we conducted ablation experiments. Specifically, we replaced the proposed Spatio-Temporal Graph Convolutional Neural Network (STGCN) with a traditional graph neural network. Additionally, in the data input module, we removed the sensor embedding to evaluate the impact of sensor embedding on learning data features. Similarly, we also experimented with a model without the attention mechanism. The experimental results are shown in Table 5.

**Table 5:** Performance Comparison of Models.

Models	Precision	Recall	F1-Scores
Ours	99.32	71.21	0.83
-STGCN	94.31	65.43	0.75
-Sensors Embedding	93.21	63.21	0.74
-Attention	89.31	58.54	0.64

In summary: (1) Replacing the proposed Spatio-Temporal Graph Convolutional Neural Network (STGCN) with a traditional graph neural network resulted in a decrease of approximately 5% in precision, 6% in recall, and 0.08 in F1-score. This clearly demonstrates the significant improvement in model performance achieved by the proposed spatio-temporal graph convolutional architecture, particularly in real-world scenarios.

(2) Removing the sensor embedding led to a decrease of approximately 6% in precision, 8% in recall, and 0.09 in F1-score. This likely indicates the reduced ability of the model to capture the relationships between sensors without the embedding, highlighting the importance of the sensor embedding in learning the interdependencies among sensors.

(3) Eliminating the attention mechanism resulted in a decrease of approximately 10% in precision, 13% in recall, and 0.19 in F1-score. This reduction is the most significant among the ablation experiments, indicating that the absence of the attention mechanism makes it challenging for the model to capture the temporal and spatial dependencies in the data. This can be attributed to the attention mechanism's ability to extract global features.

These ablation experiments demonstrate that the spatio-temporal graph convolutional neural network architecture, sensor embedding, and attention mechanism in the proposed model contribute to improving the overall accuracy of anomaly prediction. Furthermore, it explains why the proposed model outperforms the baseline.

### 3.5 OOD analysis

This section primarily focuses on the interpretability of the model. Firstly, we discuss the interpretability of sensor embedding. The t-SNE plot of the sensor data embedding in the SCTDataset is shown in Fig. 1. The dashed circular region highlights a local cluster of CO sensors, indicating that these sensors placed at different locations within the tunnel exhibit similar measurements. Therefore, in this spatial representation, the similarity between the data acquisition patterns of the sensors can be represented by the similarity between sensor embeddings, i.e., clustering.

**Figure 3:**
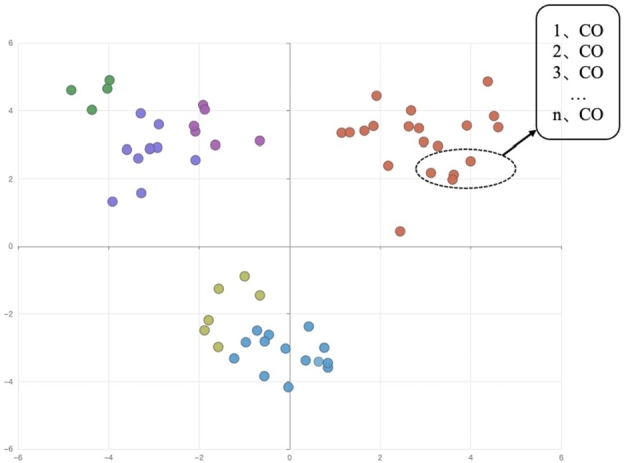
Information clustering representation of sensor influencing factors.

To validate this observation, we consider the influencing factors for sensor measurements, such as body temperature, heart rate, tunnel ambient temperature, tunnel ambient humidity, CO concentration, and chlorine concentration. These factors are selected using the Analytic Hierarchy Process (AHP). We assign six different colors to represent the six types of sensors mentioned above. Then, we project the representation vectors of the sensors into a two-dimensional space to visualize their clustering patterns, which are shown in Fig. 3. Similar representations are clustered together, which further confirms that sensors of the same type exhibit similar behavior patterns in measuring gases. Therefore, this representation effectively demonstrates the similarity between sensors.

**Figure 4:**
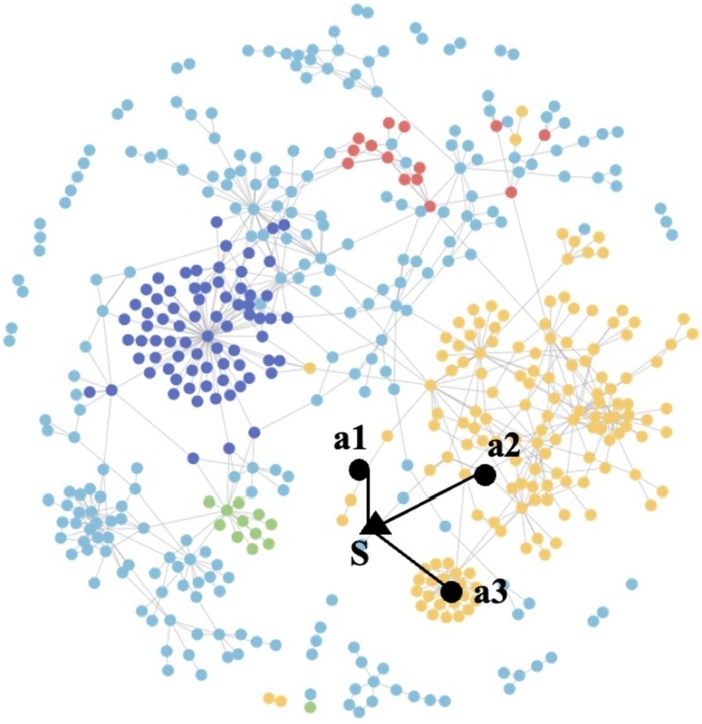
Visualization of sensor node relationships.

The main focus of this study is how to perform safety warnings for construction workers inside tunnel excavations. Visualizations were employed to address this issue. Each visualized node represents a different sensor node, while the edges depict the relationships between different sensor nodes. Edge attention weights, which reflect similar behaviors, are likely to be similar, while significantly divergent weights may indicate abnormal situations. In this case study, we investigated an exceptional scenario in the dataset originating from a sensor capturing a gas concentration exceeding the normal threshold. Specifically, the environmental temperature reached 47 degrees Celsius, a value far beyond the normal level. Consequently, sensor S was identified as the sensor with the highest deviation from the norm, as indicated by the black triangle on the left side of Fig. 4. The substantial deviation observed for this sensor signifies the occurrence of an anomaly.

In conclusion:

(1) The individual anomaly scores of our model contribute to localizing anomalies.
(2) The attention weights of the model aid in identifying closely related sensors.
(3) The model's predictions of expected behavior for each sensor enable us to understand how anomalies deviate from the expected patterns.

### 3.6 Web software architecture

Designing a web-based system for predicting and managing the safety status of tunnel construction workers requires consideration of several aspects:

**System Architecture Design:** It is necessary to determine the basic framework, modules, and components of the system, as well as their interactions. Typically, a frontend-backend separation architecture can be adopted, dividing the system into a frontend user interface and a backend server. The frontend user interface can be developed using web technologies such as HTML, CSS, and JavaScript, while the backend server can be implemented using popular web frameworks like Django, Spring, Flask, etc.

**Database Design:** A database needs to be designed to store personal information of construction workers, work environment data, safety status, and other related data. It is possible to use relational databases like MySQL, PostgreSQL, or NoSQL databases like MongoDB.

**Data Collection and Processing:** Various data sources such as personal information of construction workers and work environment data need to be collected and undergo data cleaning, processing, and storage. Data processing tools like pandas, NumPy in Python, or data collection software can be utilized.

**Data Analysis and Prediction:** Analyzing and predicting the collected data using AHP and anomaly detection algorithm models are necessary to identify potential safety risks and provide accurate management recommendations and decision support.

**User Interface Design:** Designing a user-friendly and easy-to-use interface is crucial so that managers can conveniently view the safety status of construction workers and promptly identify and address potential safety risks.

In summary, designing a web-based system for predicting and managing the safety status of tunnel construction workers requires comprehensive consideration of various factors, including system architecture, database design, data collection and processing, data analysis and prediction, user interface design, as well as system deployment and maintenance. This ensures the efficient, reliable, and secure operation of the system.

## 4 Conclusion

In conclusion, this paper addresses the challenge of ensuring the safety of construction workers in tunnels by proposing a novel model called Tunnel-APH-AD. By analyzing influencing factors and employing the Analytic Hierarchy Process (AHP), we identified essential factors for assessing worker safety in tunnels. Through the use of ensemble learning techniques and anomaly detection algorithms, we achieved improved safety state detection. Experimental results demonstrated that the ensemble learning model outperformed individual models, highlighting the effectiveness of combining multiple models for accurate safety warnings. This study provides scalable and scientifically generalized applications of machine learning in tunnel construction worker safety, contributing to proactive risk mitigation and worker well-being.

## CRediT authorship contribution statement

**Yuhao Cao:** Writing – original draft. **Bai Yun:** Writing – review & editing, Writing – original draft.

## Declaration of Competing Interest

The authors declare that they have no known competing financial interests or personal relationships that could have appeared to influence the work reported in this paper.

## Data Availability

The data supporting the findings of this study can be found on ScienceDB at https://doi.org/10.57760/sciencedb.11390 and https://www.scidb.cn/en/s/JziIFz.

## References

[1] Alpher F., Fotheringham-Smythe F., Gamow F. (2004). Can a machine frobnicate?. J. Foo.

[2] Alpher F., Gamow F. (2005).

[3] Hasan M., Choi J., Neumann J., Roy-Chowdhury A.K., Davis L.S. (2016). 2016 IEEE Conference on Computer Vision and Pattern Recognition.

[4] Zhao Y., Deng B., Shen C., Liu Y., Lu H., Hua X. (2017). Proceedings of the 2017 ACM on Multimedia Conference.

[5] Park H., Noh J., Ham B. (2020). 2020 IEEE/CVF Conference on Computer Vision and Pattern Recognition.

[6] Liu Z., Nie Y., Long C., Zhang Q., Li G. (2021). 2021 IEEE/CVF International Conference on Computer Vision.

[7] Lv H., Chen C., Cui Z., Xu C., Li Y., Yang J. (2021). IEEE Conference on Computer Vision and Pattern Recognition.

[8] Gong D., Liu L., Le V., Saha B., Mansour M.R., Venkatesh S. (2019). 2019 IEEE/CVF International Conference on Computer Vision.

[9] Lu C., Shi J., Jia J. (2013). IEEE International Conference on Computer Vision.

[10] Mahadevan V., Li W., Bhalodia V., Vasconcelos N. (2010). The Twenty-Third IEEE Conference on Computer Vision and Pattern Recognition.

[11] Bergmann P., Fauser M., Sattlegger D., Steger C. (2019). IEEE Conference on Computer Vision and Pattern Recognition.

[12] Luo W., Liu W., Gao S. (2017). IEEE International Conference on Computer Vision.

[13] Xu F., Wang N., Wen X., Gao M., Guo C., Zhao X. (2023). Few-shot message-enhanced contrastive learning for graph anomaly detection. https://arxiv.org/abs/2311.10370.

[14] Ilg E., Mayer N., Saikia T., Keuper M., Dosovitskiy A., Brox T. (2017). 2017 IEEE Conference on Computer Vision and Pattern Recognition.

[15] van den Oord A., Vinyals O., Kavukcuoglu K. (2017). Advances in Neural Information Processing Systems 30: Annual Conference on Neural Information Processing Systems 2017.

[16] Wu H., Xion W., Xu F., Luo X., Chen C., Hua X.S. (2023). PastNet: introducing physical inductive biases for spatio-temporal video prediction. https://arxiv.org/abs/2305.11421.

[17] Loshchilov I., Hutter F. (2017). 5th International Conference on Learning Representations.

[18] Deng H., Li X. (2022). Proceedings of the IEEE/CVF Conference on Computer Vision and Pattern Recognition (CVPR).

[19] Akcay S., Abarghouei A.A., Breckon T.P. (2018). Computer Vision - ACCV 2018 - 14th Asian Conference on Computer Vision.

[20] Yi J., Yoon S. (2020). Computer Vision - ACCV 2020 - 15th Asian Conference on Computer Vision.

[21] Hou J., Zhang Y., Zhong Q., Xie D., Pu S., Zhou H. (2021). 2021 IEEE/CVF International Conference on Computer Vision.

[22] Xu F., Wang N., Wu H., Wen X., Zhao X. (2023). Revisiting: graph-based fraud detection in sight of heterophily and spectrum. https://arxiv.org/abs/2312.06441.

[23] Li C., Sohn K., Yoon J., Pfister T. (2021). IEEE Conference on Computer Vision and Pattern Recognition.

[24] Zavrtanik V., Kristan M., Skocaj D. (2021). 2021 IEEE/CVF International Conference on Computer Vision.

[25] Roth K., Pemula L., Zepeda J., Schölkopf B., Brox T., Gehler P.V. (2021). Towards total recall in industrial anomaly detection. https://arxiv.org/abs/2106.08265.

[26] Higgins I., Matthey L., Pal A., Burgess C.P., Glorot X., Botvinick M.M. (2017). 5th International Conference on Learning Representations.

[27] Luo W., Liu W., Gao S. (2017). 2017 IEEE International Conference on Multimedia and Expo.

[28] Zhao B., Fei-Fei L., Xing E.P. (2011). The 24th IEEE Conference on Computer Vision and Pattern Recognition.

[29] Jaderberg M., Simonyan K., Zisserman A., Kavukcuoglu K. (2015). Advances in Neural Information Processing Systems 28: Annual Conference on Neural Information Processing Systems 2015.

[30] Dai J., Qi H., Xiong Y., Li Y., Zhang G., Hu H. (2017). IEEE International Conference on Computer Vision.

[31] Wu H., Wang S., Liang Y., Zhou Z., Huang W., Xiong W. (2023). Earthfarseer: versatile spatio-temporal dynamical systems modeling in one model. https://arxiv.org/abs/2312.08403.

[32] Loshchilov I., Hutter F. (2017). Fixing weight decay regularization in Adam. https://arxiv.org/abs/1711.05101.

[33] Liu W., Luo W., Lian D., Gao S. (2018). 2018 IEEE Conference on Computer Vision and Pattern Recognition.

[34] Defard T., Setkov A., Loesch A., Audigier R. (2020). International Conference on Pattern Recognition.

[35] Liu R., Lehman J., Molino P., Such F.P., Frank E., Sergeev A. (2018). Advances in Neural Information Processing Systems 31: Annual Conference on Neural Information Processing Systems 2018.

[36] Wu Z., Pan S., Long G., Jiang J., Chang X., Zhang C. (2020). Proceedings of the 26th ACM SIGKDD International Conference on Knowledge Discovery & Data Mining.

[37] Livieris I.E., Pintelas E., Pintelas P. (2020). A CNN–LSTM model for gold price time-series forecasting. Neural Comput. Appl..

[38] Gasparin A., Lukovic S., Alippi C. (2022). Deep learning for time series forecasting: the electric load case. CAAI Trans. Intell. Technol..

[39] Du S., Li T., Yang Y., Horng S.J. (2020). Multivariate time series forecasting via attention-based encoder–decoder framework. Neurocomputing.

[40] Fan C., Zhang Y., Pan Y., Li X., Zhang C., Yuan R. (2019). Proceedings of the 25th ACM SIGKDD International Conference on Knowledge Discovery & Data Mining.

[41] Elsworth S., Güttel S. (2020). Time series forecasting using LSTM networks: a symbolic approach. https://arxiv.org/abs/2003.05672.

[42] Sirisha U.M., Belavagi M.C., Attigeri G. (2022). Profit prediction using Arima, Sarima and LSTM models in time series forecasting: a comparison. IEEE Access.

[43] Khan S., Naseer M., Hayat M., Zamir S.W., Khan F.S., Shah M. (2022). Transformers in vision: a survey. ACM Comput. Surv..

[44] Li S., Jin X., Xuan Y., Zhou X., Chen W., Wang Y.X. (2019). Enhancing the locality and breaking the memory bottleneck of transformer on time series forecasting. Adv. Neural Inf. Process. Syst..

[45] Cao D., Wang Y., Duan J., Zhang C., Zhu X., Huang C. (2020). Spectral temporal graph neural network for multivariate time-series forecasting. Adv. Neural Inf. Process. Syst..

[46] Eldele E., Ragab M., Chen Z., Wu M., Kwoh C.K., Li X. (2021). Time-series representation learning via temporal and contextual contrasting. https://arxiv.org/abs/2106.14112.

[47] Kim T., Kim J., Tae Y., Park C., Choi J.H., Choo J. (2021). International Conference on Learning Representations.

